# Economic valuation of ecosystem services: application of a choice experiment approach on mount Guna services, North West of Ethiopia

**DOI:** 10.1016/j.heliyon.2021.e07164

**Published:** 2021-05-31

**Authors:** Yirga Wondifraw, Tefera Berihun Taw, Eshetie Woretaw Meried

**Affiliations:** a Environmental and Natural Resource Economics, Amhara Revenue Bureau, Bahir dar, Ethiopia; b School of Economics, University of Gondar, Gondar, Ethiopia

**Keywords:** Choice experiment, Ecosystem, Willingness to pay, Mount Guna

## Abstract

Guna Mountain is the highest point with an elevation of 4,113 m above sea level and located in South Gondar Zone. It is surrounded by Estie, Lay Gayint, Begemidir Guna, and Farta Woredas and served as a source of livelihood for the nearby communities. However, the area of Mount Guna has been still unprotected because of a lack of suitable conservation measures and weak control of inappropriate practices. Moreover, empirical investigations on the value of the mountain in terms of its ecosystem service are scanty available. This study aims at valuing ecosystem services of Guna Mountain using the choice experiment method and hence identifying farmer's choices of ecosystem service attributes. By considering farmers' preferences, watershed protection service, harvesting of medicinal plants, and water supply together with the cost of management attribute were identified as ecosystem service attributes. Primary data were collected from randomly selected respondents and the Random Parameter Logit model (RPL) was employed for estimation. Results of this model revealed that revealed that the signs of attribute levels are positive except for watershed protection service level 25%, and harvesting of a medicinal plant 20%. In addition to this, socioeconomic variables were significant such as family size and farm distance with a negative sign, education, and land size with a positive sign. The estimated MWTP from the basic model of RPL for non-monetary attribute levels were Ethiopian Birr (ETB)^1^ 8.575 and 175.526 per household per year for watershed protection service level 25% and watershed protection service level 50%. It was ETB 1.190 and 24.487 per household per year for the harvesting of a medicinal plant at 10% and harvesting of a medicinal plant at 20%. The contribution was ETB 116.868, 112.042, and 26.776 for water supply at two seasons of the year, water supply at three seasons of the year, and water supply for all seasons of the year attribute levels respectively. Valuing watershed protection was more than water supply followed by harvesting of medicinal plants attribute. To improve the quality and quantity of protection and water supply service, the government and farmers should work together.

## 1 Introduction

Ecosystem services’ demand has been increasing rapidly as populations and environmental dwindling, and awareness of the community increase. Then, valuation is a precondition in managing these services (Millennium Ecosystem Assessment, 2006). Ecosystem provides crucial role of provision, regulation, cultural and other for numerous stakeholders involved in the surroundings. A wide range of stakeholders are overplaying on many of the ecosystem services which may leads to conflicting interests and, the over-exploitation of some services (forest, water system, biodiversity and so on) at the cost of others. This in turn leads to the total degradation of ecosystem services which may affect the wellbeing of the upcoming generation, and other services. Ecosystem can be categorized as an environmental asset like any other assets of capital. It provides a flow of services overtime in which the consumer may reap a benefits. In this way, attaching monetary valuing for the service obtained plays a paramount significant roles in policy and decision making process, (Ibid).

Mount Guna afro alpine ecosystem is known to be the home of nationally and globally important biodiversity's and the source of many rivers that drain to the three basins, namely Abay, Tekeze, and Lake Tana basin. Besides, Mount Guna is the origin of the Rib, Gumara and many other rivers which flow down to the Lake Tana catchment area. A total of 4,615 ha around Mount Guna is currently administered under formal protection with the full mandate of the Regional Council and Local Communities. Species such as Fiestuka, Erica and Thyme which were disappearing have now started regenerating and lives a variety of bird and some wild animals have been reported by the locals. Millions of the nearby residents directly depend on the catchment and its resources for their livelihoods (Ibid).

Conservation of Mount Guna provides raw materials, regulation services, recreational opportunities for human wellbeing with its outstanding beauty, diverse attractions and great tourism potentials and, development or promotion of cultural, historical and natural heritages. It is better to sacrifice our wellbeing to preserve the environment as it is natural capital, one of economy's vital assets that give various purposes. Hence, ecosystem services are the benefits the society gets from ecosystems. These consist of different services like food and water; regulating services including regulation of floods, drought, and disease; supporting services including soil formation and nutrient cycling; and cultural services such as recreational and other important benefits (Costanza and d’Arge, 1997).

However, Mount Guna had been still unprotected area. This is because of population growth, poverty, traditional cultivation, decreasing productivity, low awareness and sense of ownership on values of wildlife and their habitats, lack of suitable conservation measure. Unless restoration, enhancement, conservation and management mechanisms to these ecosystems are implemented, multiple benefits are being lost as a result (Mekonnen, 2013).

Valuation of ecosystem services is the process of estimating the effects of changes in ecosystem services against other stuff that enhance human wellbeing. It offers a tool which improves decision makers' ability to evaluate tradeoffs among different ecosystem management systems (Millennium Ecosystem Assessment, 2006). There are various valuation methods that are currently used to measuring the respondent's willingness to pay for certain environmental changes improvement or deterioration. Out of the valuation methods, choice experiment is the yet growing method used in environmental economics. This is due to the advantage of choice experiment over contingent valuation technique in detecting potential source of biases and providing a broad base data from respondents (Hanley et al., 2001). I spite of this, comprehensive studies on valuation of the multi-functions and services of Afro-alpine ecosystem have not undertaken. As per the knowledge of the researchers, empirical findings on the economic valuation of ecosystem attributes in Mount Guna, North West Ethiopia are scanty available. Comprehensive studies on valuation of multi-functions and services of Afro-alpine ecosystem have not received much attention in general and Guna mountain range in particular. Thus, the general objective of this study was to evaluate economic value of mount Guna ecosystem services attributes using the exploited approach, choice experiment.

## 2 Description of study area

**Figure 1:**
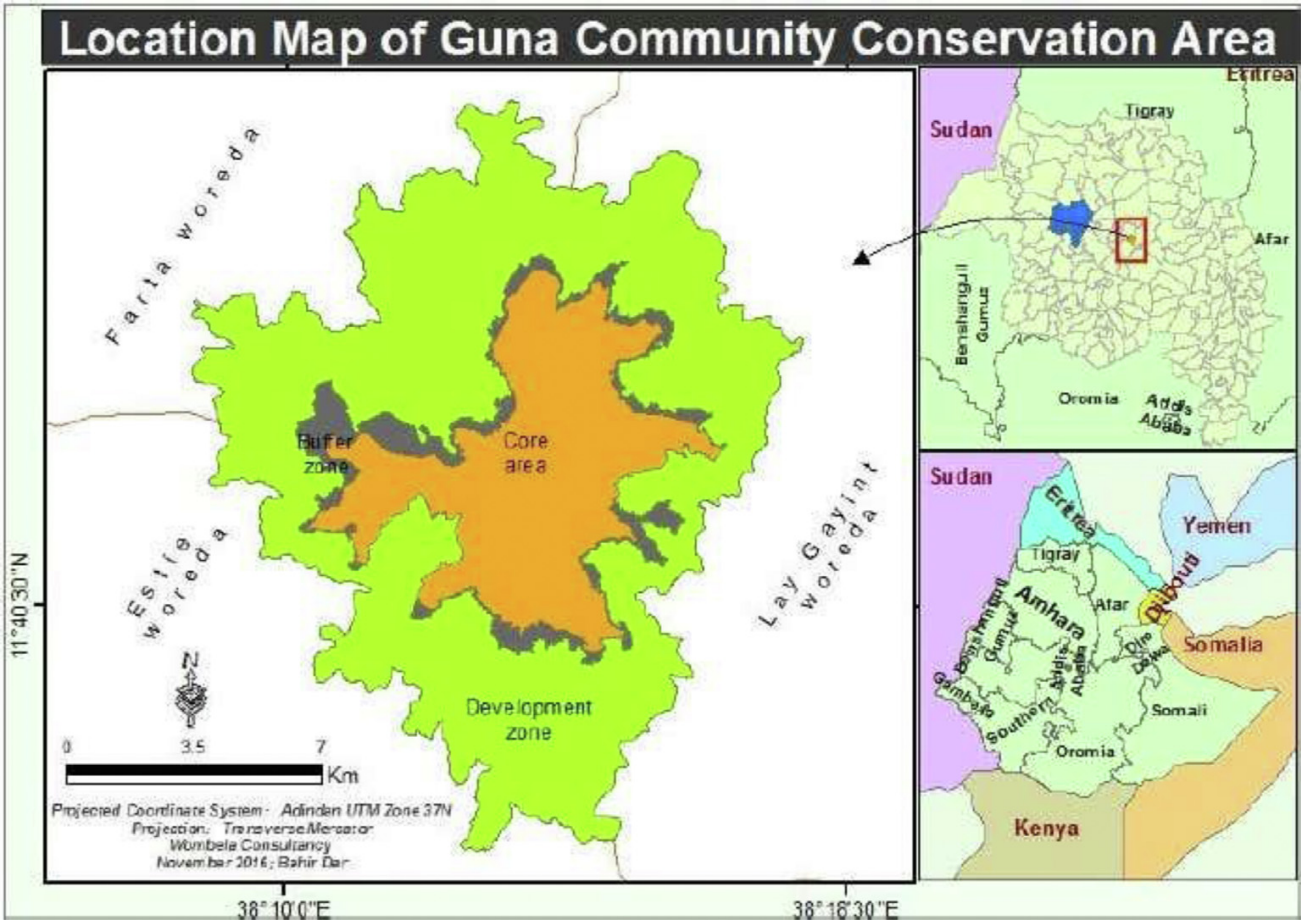
Map of mount Guna. *Source*: Bureau of culture and tourism and parks development.

Guna mountain community conservation area is located in South Gondar Zone of Amhara region, Ethiopia. It is surrounded by four Woredas including Estie, Lay Gayint, Begemidir Guna and Farta. It is the highest point in the South Gondar Zone, with an elevation of 4,113 m above sea level. Guna is found between 38°10′19.59″- 38°16′34.63″N and 11°39′48.09″- 11°45′31.61″E. The Guna mountain ecosystem area would safeguard habitats for 30 higher and small mammals, for 139 Birds and greater than 96 plant species. The Guna massif is also serving as a home for 6 and 13 endemic mammals and birds respectively (Abebaw, 2016) (see Figure 1).

## 3 Data and methods

### 3.1 Sampling and data collection method

The target population and data source of this research was heads of rural farm households whose livelihoods depend directly or indirectly on the resource of mount Guna. This is due to the fact that they are the victims and agents of ecosystem services preservation. Residence proximity to afro-alpine Mount Guna was the main rationality to randomly choose the representative Woredas and Kebeles (The lowest administrative units in Ethiopia). The range of Mount Guna afro-alpine ecosystem was the major factor to select the sample Woreda. Lay Gayint Woreda takes the lion share of Mount Guna. Due to this reason, Lay Gayint Woreda was selected as the target Woreda purposively. This Woreda has a total of 32 Kebeles and of these Kebeles, Mount Guna ecosystem was found only in four kebeles namely: Dera-Kefoye, Guna –Gedeba, Akabet, and Titra- Damot. Based on (Bennett and Blame, 2001) suggestion, the researcher allocates 50 household respondents as a sample in each Kebeles since the number of the households in each kebeles is proportionally equal. Finally, a total of 200 respondents were parts of the study, chosen using proportional simple random sampling technique (see Tables 1, 2, 3, 4, 5, 6).

**Table 1:** Example of choice set.

Attributes	Plan 1	Plan 2	Plan 3 (status quo)
Watershed protection	Improve watershed protection service by 50%	Current level of watershed protection (status quo)	Current level of watershed protection (status quo)
Harvesting of medicinal plants	Current level of medicinal plants (status quo)	Expanding the coverage of medicinal plants by 20%	Current level of medicinal plants (status quo)
Water supply	Improve the supply of water for all seasons of the year	Improve the supply of water for 2 seasons of the year	Current level of water supply (status qou)
Monetary payment (ETB)	400	300	No payment

**Table 2:** Results of socio-economic variables.

Variable	Categories	Frequency	Percentage	Mean
Sex	Male Female	176 24	88% 12%	
Age	˂24 25–29 30–49 ˃ 65	0 7 72 121	0 3.5% 36% 60.5%	53
Family size	1–3 4–6 6–9 ˃ 9	36 112 49 3	18% 46% 24.5% 1.5%	5.5
Education	Illiterate Read and write 1–8 ˃ 9	144 41 11 4	72% 20.5% 5.5% 2%	1
2Income	˂10000 10001–20000 20001–30000 30001–40000 ˃ 40000	1 63 89 43 4	5%% 31.5 44.5% 21.5% 2.0%	24,345
Farm distance	˂ 1 1–3 ˃ 3	88 99 13	44 49.5 6.5	2
Farm size	˂ 1 1–2 ˃ 2	43 78 79	21.5 39 39.5	1

**Table 3:** Farmers perception about mount Guna ecosystem degradation (multiple responses).

Degree	Causes of degradation	Responses	
		Frequency	Percentage
1^st^	Agricultural expansion	236	25.6
2^nd^	Resettlement	190	20.6
3^rd^	Climate change	182	19.8
4^th^	Deforestation	177	19.2
5^th^	Other factor	136	14.8

**Table 4:** Results of farmers preference for ecosystem services attributes.

Variable	Coefficient	Stand Error	z	Prob.|z|>Z
Nonrandom parameters in utility functions				
A_ PLAN ONE	-1.59611∗∗∗	.19585	-8.15	.0000
A_ PLAN TWO	.01631	.09708	.17	.8666
Cost of management	-.00203∗∗∗	.0005	-4.03	.0001
Random parameters in utility functions				
Watershed protection service level 25%	-.01743	.15492	-.11	. 9104
Watershed protection service level 50%	.36248∗∗	.150522	.41	.0160
Harvesting of medicinal plant attribute level 10%	-.04124	.14517	-.28	.7763
Harvesting of medicinal plant attribute level 20	.06214	.13969	.44	.6565
Water supply at two seasons of the year	.45225∗	.241151	.88	.060
Water supply at three seasons of the year	.20160	.24340	.83	.4075
Water supply all seasons of the year	.06971	.18115	.38	.700
Distns. Of RPs. Std. Deviations or limits of triangular				
NsWatershed protection service level 25%	.97070∗∗∗	.16585	5.85	.0000
NsWatershed protection service level 50%	.97070∗∗∗	.16585	5.85	.0000
NsHarvesting of medicinal plant attribute level 10%	.60585∗∗∗	.18015	3.36	.0008
NsHarvesting of medicinal plant attribute level 20%	.60585∗∗∗	.18015	3.36	.0008
NsWater supply at two season of the year	1.56923∗∗∗	.15144	10.36	.0000
NsWater supply at three season of the year	1.56923∗∗	.1514	10.36	.0000
NsWater supply all season of the year	1.56923∗∗∗	.1514	10.36	.0000
Log- likelihood function			-1318.33475	
McFadden Pseudo R-Squared			.1437905	
Nobs			36000	
Respondents			200	

Note: ∗∗∗, ∗∗, ∗ = =>Significance at 1%, 5%, 10% level.

**Table 5:** Results of extended random parameter logit model.

Variables	Coefficient	St.Error	z	Prob.|z|>Z∗
Random parameters in utility functions				
Watershed protection service level 25%	-.00963	.15549	-.06	.9506
Watershed protection service level 50%	.37255∗∗	.15171	2.46	.0141
Harvesting of medicinal plant attribute level 10%	-.05488	.14520	-.38	.7055
Harvesting of medicinal plant attribute level 20%	.04971	.13966	.36	.7219
Water supply at two seasons of the year	.42619∗	.24116	1.77	.0772
Water supply at three seasons of the year	.16598	.24878	.67	.5047
Water supply all seasons of the	.01586	.18298	.09	.9309
Nonrandom parameters in utility functions				
PLAN3	-3.03486∗∗∗	.94397	-3.21	.0013
Mgt cost	-.00204∗∗∗	.00051	-3.96	.0001
ONE_SEX1	.57486	.45793	1.26	.2094
ONE_INCOME1	-.12187D-04	.1849D-04	-.66	.5098
ONE_AGE1	.00249	.01352	.18	.8539
ONE_FAMILY SIZE1	-.12444	.08655	-1.44	.1505
ONE_LAND SIZE1	-.23289	.40205	-.58	.5624
ONE_FARM DISTANCE1	.23396∗∗	.11372 2	.06	.0396
ONE_EDUCATION1	.66373∗∗∗	.196213	.38	.0007
TWO_SEX2	-.38390	.31057	-1.24	.2164
TWO_INCOME2	-.10186D-	.1436D-04	-.71	.4781
TWO_AGE2	.00854	.00824	.04	.3000
TWO_FAMILY SIZE2	.09977	.06091	.64	.1014
TWO_LAND SIZE2	-1.09809∗∗∗	.30137	-3.64	.0003
TWO_FARM DISTANCE2	.11695	.08091	1.45	.1484
TWO_EDUCATION2	.28380∗∗	.13091	2.17	.0302
Log-likelihood function			-1112.91910	
McFadden Pseudo R-squared			.1558145	
Number of observations			3600	
Number of respondents			200	

Note: nnnnn.D-xx or D + xx => multiply by 10 to -xx or + xx.

Note: ∗∗∗, ∗∗, ∗ = => Significance at 1%, 5%, 10% level.

**Table 6:** Mean marginal WTP of improved Guna mountain ecosystem services.

Attribute levels	MWTP	95% confidence interval	
Watershed protectionat25%	8.575	-109.903	127.054
Watershed protection at 50%	175.526	34.783	316.270
Harvesting medicinal plant at 10%	1.190	-117.907	120.285
Harvesting medicinal plant at 20%	24.487	-90.665	139.639
Water supply 2 season of the year	116.868	-48.108	281.844
Water supply 3 season of the year	112.042	-39.782	263.866
Water supply all season of the year	26.766	-105.375	158.907

***Source*:** Authors Computation from Survey data, 2019

### 3.2 Design of choice experiment approach

The foundation of stated preference experiment is a survey design. The manipulation of the levels of the variables demands a particular form of a statistics to decide what manipulation to make and when to make them (David et al., 2005). The following steps were involved in the design of choice experiment.

#### 3.2.1 Description of attributes and attribute levels

To identify attributes and attribute levels the researchers used formal and informal contact with local communities to understand the perception of the locals towards the conservation area, the cause, and potential solutions of conflicts between the governments and communities. Furthermore, attributes were selected after conducting a Focus Group Discussion (FGD) with afro-alpine residents, related literature and key informants. The potential attributes that were proposed for FGD discussants were, provisioning services including food and water; regulating services consisting regulation of floods, drought, and disease; supporting services such as soil formation and nutrient cycling; and cultural services including recreational and other important benefits by assuming that other attributes of the Mount Guna ecosystem services were held constant. A thorough consultation with experts and review on a valuation of ecosystems, three non-monetary attributes, and one cost attribute were identified with their respective levels for choice experiment designation. These are watershed protection, harvesting of medicinal plants, water supply, and management cost along with the yearly charge.

A watershed protection service was leveled in three choices. That is improving watershed protection service by 25%, improving watershed protection service by 50%, and the status quo level. The harvesting of medicinal plants attribute was leveled in three levels. That is the current coverage of medicinal plants, expanding the coverage by 10% and expanding the coverage by 20%. The water supply attribute was leveled in four choices. These were one season, two seasons, three seasons, and for all seasons of the year. Last but not least, monetary attribute considers yearly payment on each household and had four levels. Its levels were 0, 100, 200, 300 and 400 each in Ethiopian Birr.

After the policy relevant attributes and their levels were identified, choice sets were construction using experimental design. The combination of different levels of the aforementioned attributes results different policy option scenario and construction of choice cards. This study deployed orthogonal fractional factorial design procedure by using Ngene 1.1.2 choice metrics statistical software. The experimental design through this software results the creation of 36 optimal choice sets. The choice set was divided in to 6 blocks, so that each respondent received 6 choice sets/cards only.

### 3.3 Method of analysis of choice experiment approach

Random utility theory formulated by Luce (1959) and Lancaster consumer choice model (Lancaster, 1966) are foundation for stated preference choice experiment approach. According to these scholars, the total utility derived from a consumption of a good is a function of the observable or random component whose values directly depend on the attributes and the other unobservable error component which is assumed to be the error terms of the resulting utility function independently and identically distributed (Louviere et al., 2000).

For an individual, the utility of choosing alternative n is a function of the attributes of the option **n**. The utility function **Unt** composed of random or observable part, **Vtn** which is in turn composed of environmental attributes of ecosystem (**Ztn)** and the socio-economic characteristics of the respondent (**Stn**), and the error component portion often that stands for unobservable random variables.

The random utility function is shown as follows

(1) Unt=V(Znt,St)+εnt

(2) Unt=Znt+St+εnt

where Unt stands for the utility of individual t for choosing the n option, Z- Indicates attributes of Guna mountain, St-indicates the socio-economic characteristics of an individual t, V***nt*** the vector of deterministic component of the latent utility that is the function of the attributes of the alternative and the socio-economic characteristics of the individual that individual ***t*** has from choosing alternative***.*** ε***nt*** is the vector of random component of the latent utility associated with option ***i*** and consumer ***t*** (Adamowicz and Boxall, 2001) which is not correlated with the deterministic part by assumption of Identically and Independently Distributed (IID).

However, it is unable to forecast respondent preferences because of the error term. Therefore, by expressing preference in terms of probabilities can be predict individual preferences. Thus, the likelihood of every respondent prefers option ***n*** in the choice set to any alternative option ***h***, can be articulated as the probability that the utility linked with option n surpass those linked with all other alternative.

(3) $$P\left(\frac{i}{\mathrm{Cn}}\right)=\mathrm{Prob}\left\{\mathrm{Unt}>Uht\right\}\;\mathrm{for}\mathrm{all}\;n\text{,}helements\;of\;C\text{, }n\forall h$$

(4) $$P\left(\frac{i}{\mathrm{Cn}}\right)=\mathrm{Prob}\left\{\mathrm{Vnt}+\varepsilon \mathrm{nt}>Vht+\varepsilon ht\right\}\;\mathrm{for}\;\mathrm{all}\;n\text{,}h\;\mathrm{elements}\;\mathrm{of}\;C\text{,}\;n\forall h$$

where, **C**n is the set of all possible alternative scenarios. To estimate Eq. (4), assumptions need be made over the distribution of the error terms. The most widely applicable assumption made is that the errors are Gumble-distrbuted (Type I Extreme value distribution) and independently and identically distributed. Depending on the assumption we put on the error term, we may come up with different models.

#### 3.3.1 The multinomial logit model (MLM)

Multinomial model is easiest and straight forward in estimating choice modeling. Under this model, the probability of selecting option is formulated as a function of attributes and socio-economic variables of the respondents (Greene and Hensher, 2003).

In order to estimate choice probabilities using MLM it is assumed that

i. The random components are independently and identically distributed (IID)
ii. The choice probability of the alternative depends only on the difference in the systematic utilities of different alternatives rather than actual values.

Based on the above assumption the probability of choosing alternative scenario multinomial model has the following expression

(5) $$\;p_{i}=\frac{exp^{\gamma Vnt}}{\Sigma exp^{\gamma th}}$$

where, γ is the scale parameter, it is impossible to identify this parameter from the data. In addition, to this the model can be estimated by maximum likelihood estimation taking the log likelihood function.

(6) $$L\mathrm{og}\;L=\overset{N}{\underset{n=1}{\sum }}\overset{n}{\underset{t=1}{\sum }}\mathrm{YtnLog}\frac{\mathrm{ex}p^{\mathrm{Vtn}}}{\sum_{t=1}^{n}\mathrm{ex}p^{\mathrm{Vtn}}}$$

Yij is an indicator variable that takes available of one if individual or respondent choose alternative n and 0 otherwise. To estimate the multinomial logit model, modeling constant known as Alternative Specific Constant (ASC) are included in the multinomial logit model.

$$\mathrm{Vnt}=\mathrm{ASSi}+\underset{M}{\sum }\mathrm{BimHim}$$

where **ASCi** is an alternative specific constant which represents the role of the unobserved source of utility for option n, **H**im is the $M^{th}$attribute value of alternative **n**. the effect of attribute on the choice set are captured by Z variables.By interacting ASC with socioeconomic variable is one of the possible mechanisms of considering individuals or respondents Heterogeneity or detect “Hessian’’ singularities.

The model can be specified in the following form.

(8) $$Vnt=ASCi+\underset{M}{\sum }B_{M}H_{M}+\sum {ϒ}_{nt}\left(ASCi*St\right)$$

where St represents the socioeconomic or attitudinal variables for individual t, and ${ϒ}_{nt}$ is the coefficient associated with the individual socioeconomic characteristic's interaction with the ASC.

#### 3.3.2 Random parameter logit model (RPL)

To avoid the problem related to the MNL model, Greene and Hensher (2003) have recommended the use of the RPL model due to due to the following advantages of the RPL over the MNL model. First, RPL is not subject to the IIA assumptions. Second, it accommodates correlation among panel observations. Third, the process unambiguously incorporates heterogeneity in taste variation across observations by permitting the model parameters to vary randomly over respondetns (Adamowicz and Boxall, 2001).

The random utility function for the random parameter logit model takes the following form:

(9) Unt=Vnt+εnt=Zi(B+nt)+Eit

where:Unt is the total utility for respondent t from choosing alternative n in the choice set. It is assumed that the utility function consists of both systematic component (Vnt) and stochastic component (εnt). The indirect utility is assumed to be a function of the choice attributeZwith parameters B(and socio economic & environmental attribute variables, if included in the model), which due to preference heterogeneity may vary across respondents by a random componentnt.

The probability that an individual ***t*** chooses alternative ***i*** from each choice set is presented as

(10) $$\;p_{i}=\frac{exp^{\gamma V\left(nt+B\right)}}{\Sigma exp^{\gamma \left(tn+B\right)}}$$

As mentioned by Birol et al. (2005), the RPL model does not require the IIA assumption, the stochastic part of utility may be correlated among alternatives and across the sequence of choices via the common influence ofnt. Moreover, RPL model is superior to multinomial logit model in terms of overall fit & welfare estimates.

#### 3.3.3 Marginal willingness to pay (part worth)

Implicit prices for ecosystem attributes are the estimations of the WTP of individuals for an improvement in the attribute of interest, given everything else is constant. Implicit prices are determined with the following formula:

(11) $$\text{Implicit price }\left(\text{Part worth}\right)=-\left(\frac{\text{βnon}-\text{market attribute of ecosystem}}{\text{β monetary attribute}}\right)$$

where, β are the estimated coefficients of the attributes.

### 3.4 Econometric specification of ecosystem choice experiment approach

In both MNL and RPL Models, the three utility functions were estimated for three alternatives: The status quo, Mount Guna Ecosystem services improvements, plan (Alternative) one and two. The basic and extended MNL models are described in the following general forms.

(12) $$\begin{array}{l} \mathrm{Ui}=b\_\mathrm{wshedp}*\mathrm{wshedp}+b\_\mathrm{harmed}*\mathrm{harmed}+b\_\mathrm{watsup}*\mathrm{watsup}+b\_\mathrm{cost}*\mathrm{cost}+\theta 1ASCi*AGE+\theta 2ASCi*FAMSZ+\theta 3ACi*\\ EDUS+\theta 4ASCi*INC+\theta 5ASCi*GNDR+\theta 6ASCi*FARDIST+\theta 7ASCi*LANDSIZE\; \end{array}$$

b-represents the coefficients associated with each of the four attributes, i.e. improvement in, watershed protection (wshedp), harvesting medicinal plants (harmed) water supply (watsup), and monetary payment (cost) respectively. ASCi = Alternative specific constant and takes the value **0** for status quo option and **1** for choose alternative one and two. Whereas, AGE stands for age, FAMS (family size), EDUS (education), INC (income), GNDR (gender), DSKM (distance from the mountain), and LANDSIZE is size of land as socioeconomic variables,θi is the coefficient of socioeconomic variables.

## 4 Results and discussion

### 4.1 Descriptive statistics

Ahead of conducting the full survey, pretesting has been conducted on a small number of respondents, 12 households who were not included latter in the main survey. From this we got a paramount significance for the appropriate modifications of any shortcoming of the designed questionnaire and as training for enumerators. The pretest result revealed that the questionnaire was very long, time demanding 65 and not written in simple word. Thus, these factors were considered before the main survey. After some adjustment, the questionnaire was made ready for the main survey. Concerning about the response rate, it was 100 %, the sampled respondents were willing and committed to take part the full field survey implying that they are concerned about the improvement in ecosystem services in their vicinity.

Further, about 88% of respondents were male and the remaining were female-headed rural households. The mean family size of the farmers or household heads was about 5.5 which are greater than the National Central Statistical Agency standard of family size 4.5. The mean age of the respondents was found to be 53. Concerning the level of education, 72% of respondents affirmed that they can't read and write.

The mean yearly income of the farmers was about 24,345 ETB which is equivalent to about 869.46428 USD (1$ = 27ETB). The average farm distance from Mount Guna was 2 km and the average size of farm land-holding sizes was one hectare per household.

Farmers were also asked about the major factors that affect the degradation of Mount Guna afro-alpine ecosystem area. They ranked that the first factor was agricultural expansion (25.6%), resettlement (20.6%), climate change (19.8%), deforestation (19.2%), and other factors (14.8%).

### 4.2 Econometric results of choice experiment

The package, LIMDEP10.0 NLOGIT 5.0 was used to estimate the basic and hybrid RPL mode. In the basic model, Mount Guna services attributes which explain individuals’ choice for different services interventions were considered, whereas, socio-economic and environmental variables were considered in the extended models.

Limdep 10 Nlogit 5.0 Econometrics software was used to analyze the quantitative data of the study. The estimates of random parameter logit model were used to identify important attributes: watershed protection, water supply, and harvesting of medicinal plants. The parameter logit model has an advantage in introducing respondents’ preference heterogeneity as independent variables in explaining the probability of choice (Greene and Hensher, 2003). In this study the researcher specified attributes and its levels such as WSHEDP 25%, WSHEDP 50%, HARMED 10%, HARMED 20%, WATSUP 2, Season WATSUP 3 seasons, and WATSUP all seasons to be randomly distributed.

The pseudo R-square is implying better explanatory power of random parameter logit model than the standard logit models. The result of the estimation revealed that the level of 2 seasons of water supply attribute level and watershed protection attribute level at 50 % retain the expected sign and significant at 10% and 5 % respectively. This result indicated respondents had been willing to share the cost of improving Mount Guna's level of attributes. The coefficients monetary attribute indicate that the inverse relationship between respondent utility and cost of improvement. The implication is that as cost of improvement increased, the respondent participation for the improvement of environmental plan would decrease.

Further, the finding of the study revealed significant negative value for the Alternative Specific Constant (ASC) for environmental plan one. This shows the utility of respondent decreases as they shift from the status quo to the alternative environmental improvement plans 1. According to Samuelsson and Zeckhouser, such kind of result might be due to status quo bias. It's a common phenomenon and supported by a lot of evidence from literature (Adamowicz et al., 1998). In this particular case, there were 46 respondents choosing the status quo, valuing the current situation more than the improved one. This could be low awareness and sense of ownership on values of wildlife and their habitat, look for additional farming and to satisfy daily food demand through extraction and consumption use of resource. In addition to that choosing among option might be complex to respondents and they might be uncertain whether they are willing to make trade-off or not. Choosing the status quo could also be considered as protest response (Adamowicz and Boxall, 2001).

The coefficients of distributions of respondent standard deviation result revealed that all parameters are random and significantly affects 1% level of significant. This implies that the coefficients of a particular levels of attributes (WSHEDP 25%, WSHEDP 50%, HARMED 10%, HARMED 20%, WATSUP 2 seasons, WATSUP 3 seasons, WATSUP all seasons) on the chosen alternative various across the individuals.

The log likelihood function and the pseudo R^2^ values indicated that this model fits better than the standard CLM. The basic and hybrid RPL models have an explanatory power of 14% and 16% respectively. Therefore, the model with interaction best explained the data from sample respondents and this coefficient is used for interpretation.

The extended random parameter logit model result from Table 4.4 revealed that farm distance and education could positively affect the probability of choosing environmental plans 1 at 5% and 1% level of significant respectively. The implication is that educated household had a better understanding of the benefits of ecosystem improvements. Moreover, land size with negative coefficient significantly affected the probability of choosing environmental plan 2 at 5% level of significant which suggested that as the size of the farmers owned cultivated land increases ecosystem improvement will decreases.

The socio-economic variables age, sex and income implied that the involvement of these variables were not a significant factor in affecting the probability of choosing the improved plans. But the coefficient of interaction of ASC with years of education was positive and significant. This indicates that as years of education increases, the probability of choosing improve environmental plan increases, ceteris paribus. In addition to this, the coefficient of interaction of ASC with sex is positive which implies that the probability of choosing the improvement option was higher for male as compared to female other things being constant.

The coefficient of family size was not expected to be negative for plan 1. The negative coefficient of land size was not expected for the two environmental plans but significant for plan 2 implying that as land size increases, the probability of choosing environmental plan decreases. The coefficient of the watershed attribute was greater than that of water supply attribute and harvesting of medicinal plant which implies that farmers give more value to level of watershed protection attribute.

#### 4.2.1 Estimation of marginal willingness to pay (MWTP)

Implicit prices (MWTP) for ecosystem attributes are the estimations of the WTP of respondents for an improved in the attribute of interest, given everything else is constant. Implicit prices are determined at the ratio of the coefficients of the attributes of Guan mountain afro-alpine ecosystem in random parameter logit model to the estimated coefficients of the monetary attribute. Accordingly, respondents’ marginal WTP for an environmental improvement plan for watershed protection service 25% and watershed protection service 50% is 8.575 and 175.526 ETB respectively.

Harvesting medicinal plant attribute plan 10% and 20% WTP for the improvements of harvesting of medicinal plant attribute is ETB 1.190 and birr 24.487 respectively, For the water supply attribute of Guna Mountain respondents marginal WTP for environmental improvement plan for two seasons of the year, three seasons of the year and all seasons of the year is ETB 116.868, 112.042, and 26.766 respectively. The MWTP of water supply all seasons of the year level result is low as compared to two seasons of the year and three seasons of the year. The result was not surprising because this might be choosing among option might be complex to respondents and they might be uncertain whether they are willing to make trade–off or not. In closing, respondents give priority for watershed protection attribute environmental plan 50% followed by water supply for two seasons of the year.

## 5 Conclusions

The main objective of this study was to investigate the preferences and WTP of households for enhanced Mount Guna Range Ecosystem Services using a choice experiment approach. The choice experiment data was collected from 200 rural farm households residing in North West of Ethiopia. In addition to the basic random parameter logit and the hybrid model was employed. Results from both models showed that sampled households were willing to share the cost of improving Mount Guna ecosystem services attributes.

Further, some socioeconomic variables were found to be a significant determinant in influencing respondents’ choice of improved interventions. Higher yearly marginal willingness to pay of respondents for the environmental improvement plans of the specified attributes and its levels suggested as a remedy to solve the degradation of the Mount Guna ecosystem attributes and its levels by creating other options to the farmers whose livelihood is directly depend on the Mount Guna.

## 6 Policy implications and directions to future researchers

Based on the finding of the study, the following policy implications are recommended. Large preference heterogeneity is found within respondents in the study area. Therefore, decision makers who are interested to get efficient ecosystem services conservation and improvements should consider this preference heterogeneity before developing suitable plan in Mount Guna. The implication of the community contribution for levels of Mount Guna quality improvement and options were that the service provider can create income from the society to promote ecosystem services conservation and cuts the cost of handling its improvement, and then the divergence between the demand and the supply of the Mount Guna ecosystem service can be settled.

Lastly, the issue related to the methodology used by the study indicated that CEA can be fruitfully used in the context of ecosystem services valuation. Through vigilant identification of relevant attributes, appropriate construction of the efficient choice sets, specification of models, the method can effectively be used in identifying individuals preferences for proposed policy interventions and then forward policy relevant information about enhanced environmental services. The methodology is anomalous yet and study need envisage this method to examine individual's preferences for non marketed environmental goods and services using Latent Class Logit Econometric Model too.

## Declarations

### Author contribution statement

Yirga Wondifraw: Conceived and designed the experiments; Performed the experiments; Analyzed and interpreted the data.

Tefera Berihun Taw & Eshetie Woretaw Meried: Analyzed and interpreted the data; Contributed reagents, materials, analysis tools or data; Wrote the paper.

### Funding statement

This research did not receive any specific grant from funding agencies in the public, commercial, or not-for-profit sectors.

### Data availability statement

Data will be made available on request.

### Declaration of interests statement

The authors declare no conflict of interest.

### Additional information

No additional information is available for this paper.

## Footnotes

2 During the study period, 2019: 1$ = 27 Ethiopian Birr (ETB).
